# Challenges and Implications for Menopausal Health and Help-Seeking Behaviors in Midlife Women From the United States and China in Light of the COVID-19 Pandemic: Web-Based Panel Surveys

**DOI:** 10.2196/46538

**Published:** 2024-01-26

**Authors:** Bobo Hi Po Lau, Catherine So Kum Tang, Eleanor Holroyd, William Chi Wai Wong

**Affiliations:** 1 Department of Counselling and Psychology Mrs Dorothy Koo and Dr Ti Hua Koo Centre for Interdisciplinary Evidence-Based Practice and Research Hong Kong Shue Yan University Hong Kong China (Hong Kong); 2 Faculty of Health and Environmental Sciences Auckland University of Technology New Zealand New Zealand; 3 Department of Family Medicine and Primary Care, School of Clinical Medicine Li Ka Shing Faculty of Medicine The University of Hong Kong Hong Kong China (Hong Kong)

**Keywords:** menopause, help-seeking, health beliefs, telehealth, COVID-19, women, menopausal health, women's health, online survey, health beliefs, awareness, digital health, symptom management, health education

## Abstract

**Background:**

The global population of women of menopausal age is quickly increasing. The COVID-19 pandemic has led to an accelerated increase in the use of telehealth services, especially technological solutions targeting women’s health. Understanding the factors behind midlife women’s help-seeking behaviors amidst the pandemic will assist in the development of person-centered holistic telehealth solutions targeting menopausal and postreproductive health.

**Objective:**

This study aimed to compare the factors underlying help-seeking for menopausal distress among midlife women in the United States and China.

**Methods:**

We conducted 2 web-based panel surveys in the United States using Amazon Mechanical Turk and in China using Credamo between July and October 2022. A total of 1002 American and 860 Chinese women aged between 40 and 65 years took part in the survey. The survey was designed based on the Health Belief Model with questions related to their menopausal knowledge, perceived severity of menopausal symptoms, perceived susceptibility to menopausal distress, perceived benefits of help-seeking, perceived COVID-19– and non–COVID-19–related barriers against help-seeking, self-efficacy, and motivation to seek help. Structural equations models were fitted for the data using full information maximum likelihood to manage missing data.

**Results:**

Knowledge was not directly related to help-seeking motivation in both samples. Among the Chinese sample, knowledge was negatively related to perceived severity but positively related to COVID-19–related barriers; in turn, higher perceived severity, benefits, COVID-19–related barriers, and self-efficacy and lower non–COVID-19–related barriers were related to more motivation to seek help. In the US sample, knowledge was negatively related to perceived severity, susceptibility, benefits, barriers (COVID-19– and non–COVID-19–related), and self-efficacy; in turn, higher self-efficacy, COVID-19–related barriers, and benefits were associated with more help-seeking motivation. The factors explained 53% and 45.3% of the variance of help-seeking motivation among the American and Chinese participants, respectively.

**Conclusions:**

This study revealed disparate pathways between knowledge, health beliefs, and the motivation for help-seeking among American and Chinese midlife women with respect to menopausal distress. Our findings show that knowledge may not directly influence help-seeking motivation. Instead, perceived benefits and self-efficacy consistently predicted help-seeking motivation. Interestingly, concern over COVID-19 infection was related to higher help-seeking motivation in both samples. Hence, our findings recommend the further development of telehealth services to (1) develop content beyond health education and symptom management that serves to enhance the perceived benefits of addressing women’s multidimensional menopausal health needs, (2) facilitate patient–care provider communication with a focus on self-efficacy and a propensity to engage in help-seeking behaviors, and (3) target women who have greater midlife health concerns in the postpandemic era.

## Introduction

The population of women of menopausal age is increasing. According to the United Nations, women aged 50 years or older constitutes 26% of the global female population, compared to 21% 10 years ago [1]. Globally, it is predicted that the population of midlife women will reach 1.65 billion by 2050, from about 980 million in 2020, suggesting a burgeoning global demand for menopausal health care. The European Menopause and Andropause Society therefore proposed a Healthy Menopause [2] framework that links menopausal health to the World Health Organization’s healthy aging framework [3] to empower women to make salutary choices for their postreproductive health through personalized interdisciplinary care. Although menopause is often perceived as having ambiguous temporal boundaries and requiring broad population-based interventions, studies across cultures have revealed discrepancies in the understanding of the menopause experience between women in premenopapuse and postmenopause, pointing to insufficient knowledge and understanding together with inaccurate expectations [4-6].

Midlife women often manage their symptoms through self-initiated lifestyle modifications, such as relaxation, healthy diet, regular exercises, and supplements, or simply tolerating them; yet, they tended to be conservative with seeking medical help and associated services [7-11]. A study reviewing medical records in the United States found that while most menopausal women eventually saw doctors for their worrisome symptoms [12], 40% and 13% of them had neither medication nor any therapeutic interventions documented, respectively, and about 50% of women delayed seeking medical help for more than 6 months after symptom onset. In a recent study in Shanghai, China, only 36.8% of women who had menopausal symptoms went to see a doctor, and most commonly went to internal medicine, rather than gynecology or a specialized menopausal service [13]. Coupling with the patients’ lack of knowledge about menopause and associated care options, a substantial gap in professional education may lead health care professionals to either feeling uncomfortable or less-than-competent in discussing menopause, lacking a holistic perspective about the multidimensional distress of their patients [14].

The COVID-19 pandemic accelerated the rapid rise and accompanying advances in telehealth services [15], which was documented to have increased 38-fold in the total claims volume in the United States in late 2020 when compared to early 2020 [16], with psychiatry and endocrinology services ranked numbers 1 and 3, respectively. Similarly, in China alone, there has been a 900% increase in users and 800% increase in telehealth visits by new users from December 2019 to January 2020, shortly after the emergence of the COVID-19 pandemic [17]. Reflecting on the first 2 years of the COVID-19 pandemic, a global survey with 293 professionals in different fields revealed increased confidence in telehealth [18]. With respect to improving perimenopausal and postmenopausal women’s well-being, Menotech*,* as a branch of Femtech that encompasses primarily smartphone apps and telemedicine platforms [19-21] aimed at managing symptoms, hormonal replacement therapy, and triaging low-risk women for regular checkups [22], has flourished in recent years in North America, Europe, and Australia. The increased usage of telehealth presents both opportunities and barriers for midlife women seeking health education and care, underscoring the need to examine the related influencing factors from a cross-cultural perspective.

Both China and the United States witnessed a clear spike in telehealth use during COVID-19 outbreaks [23,24]. Both countries also saw marked increases in their population of women entering menopause in recent years. In China, the total number of women in menopause has already exceeded 200 million and is expected to reach 280 million by 2030 [25]. In the United States, about 1.3 million women are entering menopause annually, with over 51 million women going through or having gone through the transition at present [26,27].

Menopause is a universal phenomenon that ensues similar long-term health consequences across populations, yet with geographical differences in the nuanced experiences of its symptoms [13,28]. Palacios et al [29] found that Asian women reported a lower age of onset (42.1-49.5 years) than their North American counterparts (50.5-51.4 years), but a similar prevalence of overall symptoms (36%-50% in North America and 22%-63% in Asia). Chinese women also tend to experience less concern with vasomotor symptoms, such as hot flashes, which are more prevalent and perceived as more embarrassing for North American women, but report more irritability and emotional distress instead [30]. In general, Asian women are likely to endorse more nonmedicalized views toward menopause, seeing it as a natural progression of life, yet perceive greater difficulties in disclosing and discussing their symptoms with their families, peers, and health professionals. Compared to their Western counterparts, they have been shown to enlist a repertoire of both Western and traditional Asian coping methods to manage with their physical and emotional symptoms [31]. The use of Chinese medicine to manage menopausal symptoms has been such an established practice among Chinese women that evidence-based Chinese medicine clinical practice guidelines are already available [32].

The purpose of menopausal health care is 2-fold—relieving the multidimensional distress associated with menopause as well as nurturing holistic health for the postreproductive lifespan [33]. With the life expectancy of women in industrialized countries reaching over 80 years and large populations of women facing the onset of menopause at about 50 years of age, understanding the factors behind menopausal help-seeking is important for fostering midlife women’s health and well-being. In this light, this study used web-based survey panels to explore the differences in help-seeking motivation and the associated factors among midlife women in the United States and China during the pandemic.

## Methods

### Design and Sampling

This study involved 2 web-based surveys with samples drawn from the United States and China. The US data were collected from Amazon Mechanical Turk (MTurk), while those from China were collected from Credamo Inc. The 2 platforms were chosen considering they are popular and well-established in conducting survey research in their respective countries at the time of data collection, and that they support a similar range of question types, participant screening procedures, and arrangements for distributing incentives. For MTurk, Robinson et al [34] reported that the panel included about 226,500 individuals based in the United States, while Credamo Inc declared over 3 million individuals on their panel based in China as of January 2023 [35]. The advantages of using web-based panels include efficient screening for participants with the desired demographic characteristics out of a vast pool, as well as ensuring that participants have experience filling in web-based surveys to safeguard data quality. In this study, which was conducted against the backdrop of the increase in telehealth use during the COVID-19 pandemic, we were particularly interested in the help-seeking motivation of midlife women who would be potential patrons of telehealth. For both samples, the inclusion criteria were being female and aged between 40 and 65 years, which was the normative window for menopausal transition ±5 years [36,37]. To safeguard data quality, we requested participants to have a HIT approval rate of ≥98% on Amazon MTurk or a historical approval rate and credibility score of ≥80% on Credamo Inc. These benchmarks were recommended by the respective platforms. On the 2 panels, the surveys were advertised and made available to eligible participants only. Informed consent was collected via the internet prior to the surveys. Participants were reimbursed for their participation at nominal prices according to the recommendations of the panels and handled by the panels.

### Ethical Considerations

Ethical approval was obtained from the Human Research Ethics Committee of Hong Kong Shue Yan University (HREC 22-04(1)).

### Instruments

Participants’ knowledge about menopause was evaluated on a 23-item scale. The first 22 items were adopted from Noroozi et al [38] and Gebretatyos et al [39]. These items covered aspects including age and factors affecting the onset and symptom severity, health impacts, and symptoms. An item on hormonal replacement therapy was added as it frequently appears on surveys about menopausal attitudes and knowledge [5,40,41]. Participants scored 1 for a correct answer and the total score ranged from 0 to 23. Perceived severity of menopausal distress was measured by the Greene Climacteric Scale [42], which has 21 items covering physical, psychological, vasomotor, and sexual symptoms. Participants were instructed to answer on a scale from 0 (not at all) to 3 (extremely) on how bothered they were by these symptoms. The scale score ranged from 0 to 63 with good reliability (US: 0.93; China: 0.95). Items of perceived susceptibility, perceived benefits, perceived barriers, self-efficacy, and motivation to seek suitable help for menopausal needs were constructed with reference to the Champion’s Health Belief Model Scale [43] (Table 1 and Textbox 1). The items were face-validated by an expert group comprising 2 general practitioners, a gerontologist, experts in women’s health and mental health, and a medical social worker. Participants were asked to indicate their agreement on a Likert scale from 1 (strongly disagree) to 7 (strongly agree). We examined the perceived COVID-19–related barriers (ie, concerns about contracting COVID-19 from the clinic and spreading it to family members) and perceived non–COVID-19–related barriers (ie, financial, time, and informational barriers and embarrassment from help-seeking) separately. Understanding midlife and older women’s concerns about contracting COVID-19 from medical clinics, separating these 2 categories of barriers was intended to support finding new ways of providing menopausal services, such as telehealth adaptations during a pandemic like the COVID-19 pandemic.

**Table 1:** Internal consistency of items assessing perceived susceptibility, perceived benefits, and perceived barriers for menopausal distress.

Scale	Items, n	Cronbach α	
		United States	China
Perceived susceptibility	2	.84	.83
Perceived benefits	2	.81	.81
Perceived COVID-19–related barriers	2	.90	.93
Perceived non–COVID-19–related barriers	7	.94	.85

**Items for perceived susceptibility, perceived benefits, perceived barriers, self-efficacy, and motivation to seek suitable help for menopausal distress.**

Perceived susceptibility

- I am likely to suffer from the negative physical impacts of menopause.
- I am likely to suffer from the negative psychosocial impacts of menopause.

Perceived benefits

- Seeing a doctor/therapist will help me cope more effectively with my menopause.
- Seeing a doctor/therapist will reduce the negative impacts of menopause on me.

Perceived COVID-19–related barriers

- I have been afraid of catching COVID from attending medical care/a clinic.
- I have been worried about passing COVID to my loved ones from attending medical care/a clinic.

Perceived non–COVID-19–related barriers

- It has been hard for me to pay for the care for my menopause.
- I don't know where to get help for my menopausal symptoms.
- Talking about menopause with a care professional is too embarrassing.
- I don't have time for attending medical care or therapies for my menopausal symptoms.
- I have more important problems to attend to other than my menopause.
- I don’t have the encouragement I need from my loved ones to attend to my menopausal distress.
- I did not get care for my menopausal distress as I don't know what the doctor/therapist will do to help.

Self-efficacy

- I am confident that I will get the help I need for my menopausal health needs.

Motivation

- I am motivated to get suitable help for my menopausal health needs.

### Statistical Analysis

Independent *t* tests were used to compare the factors affecting the motivation to seek help across the 2 samples. A structural equations model (SEM) was used to explore the associations among variables according to the Health Belief Model [44]. A model was fitted for each sample, since there was no prior study suggesting either identical or distinctive patterns of associations among the variables. The model has been fitted with the built-in SEM function of JASP (ver 0.16.4.0, The JASP Team) based on the *lavaan* R package, and the missing data were handled by full information maximum likelihood [45]. Instead of imputing or replacing the missing data, all the data in the sample were used to obtain the population parameters that maximize the likelihood functions. A satisfactory model fit was indicated by a nonsignificant result on the chi-square test, comparative fit index (CFI), and Tucker-Lewis Index (TLI) of over 0.95, as well as a root-mean-square error of approximation (RMSEA) and standardized root-mean-square residuals (SRMR) of 0.08 or less [45,46]. Since a large sample size tends to yield a significant chi-square test result regardless of the model fit, we still accepted the model if it fulfilled the last 4 criteria but with a significant chi-square test result. All tests used *P*<.05 to indicate statistical significance.

## Results

The sample characteristics are presented on Table 2. The US sample had 1002 women (mean age 48.0, SD 6.3 years) collected in 6 days in late September 2022, while the Chinese sample had 860 women (mean age 48.6, SD 5.5 years) collected on 3 separate dates in July and October 2022. Most participants had tertiary education (US: n=909, 90.7%; China: n=567, 65.9%), were married (US: n=852, 85.4%; China: n=835, 97%), and had full-time occupations (US: n=805, 80.8%; China: n=662, 77%). The median income of the US participants was between US $50,000 and US $75,000 per annum (n=382, 38.1%) and the participants were predominantly White (n=835, 83.3%). The median income of the Chinese sample was between ¥15,001 (US $2104.82) and ¥20,000 (US $2806.24) per month (US $23,078-$30,769 per annum; n=217, 25.2%). As for their menopausal statuses, 53.2% (n=533), 35.8% (n=359), and 11% (n=110) of US participants were premenopausal, postmenopausal, and perimenopausal, respectively, while the respective ratios were 37.3% (n=321), 31.1% (n=267), and 31.6% (n=272) for Chinese participants. While the number of self-reported chronic illnesses were comparable across the 2 samples, US participants were more likely to use all categories of health care services except for Chinese medicine and complementary and alternative therapies, for which Chinese participants reported greater use.

A measurement model was fitted for the 4 subscales with self-created items (perceived susceptibility, perceived benefits, perceived COVID-19–related barriers, and perceived non–COVID-19–related barriers) using confirmatory factor analysis. Satisfactory fit for 4 correlated latent factors was reported for both samples (US: *χ*^2^_59_=128.8, *P*<.001; CFI=0.99; TLI=0.99; RMSEA=0.037; SRMR=0.017; China: *χ*^2^_59_= 203.9, *P*<.001; CFI=0.96; TLI=0.97; RMSEA=0.055; SRMR=0.041).

The US sample scored lower than the Chinese sample on menopausal knowledge, perceived severity, perceived benefits, self-efficacy, and motivation, but higher on perceived susceptibility and perceived barriers (Table 3). All *t* statistics were statistically significant (*P*<.001).

Several demographic variables were associated with motivation to seek help. In the US sample, higher motivation was related to lower age (*r*=–0.79, *P*=.02), having a full-time occupation (t_914_=4.59, *P*<.001), being affiliated to a religion (t_898_=6.87, *P*<.001), and being married (t_913_=8.42, *P*=.03). Help-seeking motivation was also related to menopausal state (*F*_2,916_7.37, *P*=.001), with significantly higher motivation to seek help among women in premenopause rather than those in postmenopause (*P*<.001), and with those in perimenopause in between the other 2 states. Motivation to seek help was not significantly related to education, income, ethnicity, or the number of chronic illnesses.

In the Chinese sample, higher motivation was related to higher income (*r*=0.181, *P*<.001), having a full-time occupation (t_833_=3.758, *P*<.001), and being married (t_833_=2.168, *P*=.03). Motivation to seek help was also associated with menopausal state (*F *_2,832_=3.096, *P*=.046), with the highest motivation among women in perimenopause, followed by those in postmenopause (*P*=.04), and then those in premenopause (*P*=.03). Motivation was not significantly related to age, education, the presence of a religious affiliation, or the number of chronic illnesses.

**Figure 1:**
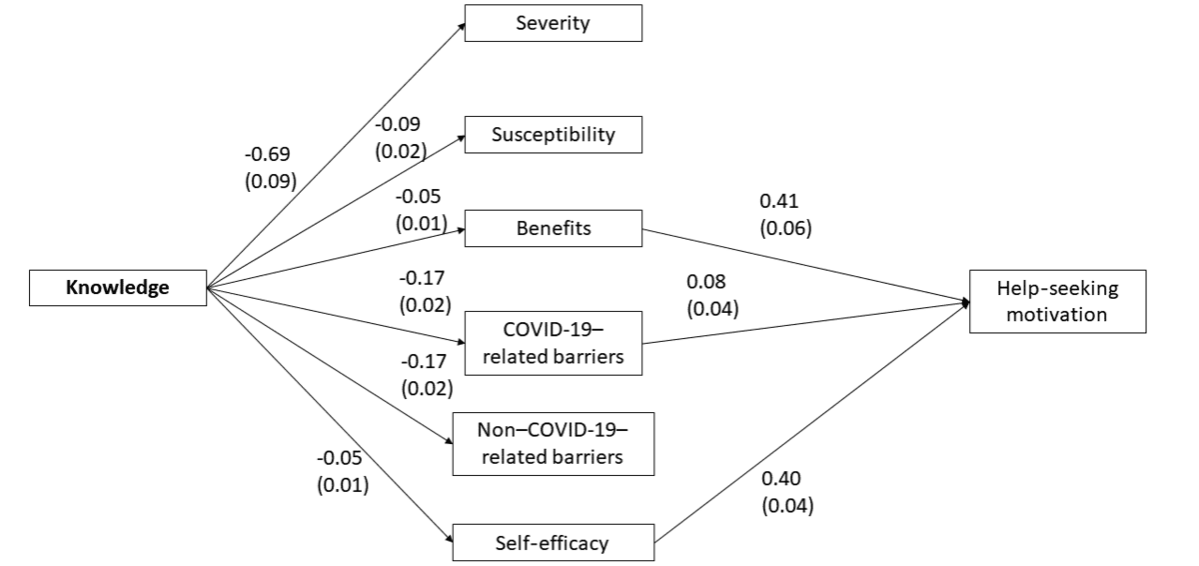
Significant paths for the structural equations model with the US sample (N=1002). The numbers denote the unstandardized estimates and the standard errors are in brackets.

SEM was used to explore the pathways between knowledge, health beliefs, and the motivation to seek help. The overall fit was satisfactory for the US model (*χ*^2^_95_=320.48, *P*<.001; CFI=0.99; TLI=0.98; RMSEA=0.038, *P*>.99; SRMR=0.018) despite the significant chi-square test result (Figure 1). The model accounted for 53% of the variance of motivation. Surprisingly, higher knowledge was related to lower perceived susceptibility, perceived benefits, perceived COVID-19– and non–COVID-19–related barriers, and even self-efficacy to seek help. Higher perceived benefits, perceived COVID-19–related barriers, and self-efficacy were related to higher motivation to seek help in American participants.

**Figure 2:**
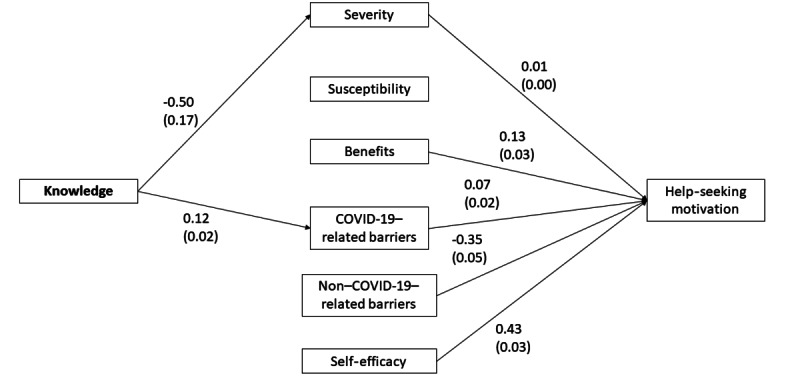
Significant paths for the structural equations model with the Chinese sample (N=860). The numbers denote the unstandardized estimates and the standard errors are in brackets.

The overall fit was also satisfactory for the Chinese model (*χ*^2^_95_=320.90, *P*<.001; CFI=0.96; TLI=0.95; RMSEA=0.053, *P*=0.24; SRMR=0.037) despite the significant chi-square test result (Figure 2). Inspection of the regression paths revealed that menopausal knowledge was associated with higher perceived COVID-19–related barriers and lower perceived severity. Higher perceived severity, perceived benefits, perceived COVID-19–related barriers, self-efficacy, and lower perceived non–COVID-19–related barriers were related to higher motivation to seek help. The model explained 45.3% of the variance of motivation. In both the United States and China models, knowledge was not significantly associated with motivation to seek help. Table 4 illustrates the results of the SEM with the United States and China data.

**Table 2:** Sample characteristics.

Variables		United States (n=1002)	China (n=860)
Age (years), mean (SD)		48.0 (6.3)	48.6 (5.5)
**Education, n (%)**			
	Primary or below	7 (0.7)	16 (1.9)
	Secondary	86 (8.6)	277 (32.2)
	Tertiary or above	909 (90.7)	567 (65.9)
**Marital status, n (%)**			
	Married	852 (85.4)	835 (97)
	Single	81 (8.1)	10 (1.2)
	Divorced or separated	65 (6.5)	15 (1.8)
**Occupation, n (%)**			
	Full-time occupation	805 (80.8)	662 (77)
	Others	191 (19.2)	198 (23)
**Religious affiliation, n (%)**			
	Yes	906 (92.4)	101 (11.7)
	No	75 (7.7)	759 (88.3)
**Income per annum (US $), n (%)**			
	0-24,999	61 (6.1)	N/A^a^
	25,000-49,999	162 (16.2)	N/A
	50,000-74,999	382 (38.1)	N/A
	75,000-99,999	170 (17.0)	N/A
	100,000-124,999	107 (10.7)	N/A
	125,000-149,999	57 (5.7)	N/A
	150,000-174,999	30 (3.0)	N/A
	180,000-199,999	17 (1.7)	N/A
	≥200,000	10 (1.0)	N/A
**Income per annum (US $), n (%)**			
	0-8,353	N/A	55 (6.4)
	8,355-16,707	N/A	158 (18.4)
	16,708-25,060	N/A	122 (14.2)
	25,061-33,414	N/A	217 (25.2)
	33,415-41,767	N/A	119 (13.8)
	41,768-50,120	N/A	61 (7.1)
	50,122-58,474	N/A	47 (5.5)
	58,475-66,827	N/A	34 (4.0)
	66,828-75181	N/A	17 (2.0)
	75,182-83,534	N/A	11 (1.3)
	≥83,535	N/A	19 (2.2)
**Race^b^, n (%)**			
	White	835 (83.3)	N/A
	Black	49 (4.9)	N/A
	Asian	97 (9.7)	N/A
	Others	21 (2.1)	N/A
**Menopausal state, n (%)**			
	Regular menses in last 12 months (premenopausal)	533 (53.2)	321 (37.3)
	Menses stopped in last 12 months (postmenopausal)	359 (35.8)	267 (31.1)
	Less than 3 menses or irregular periods in the last 12 months (perimenopausal)	110 (11)	272 (31.6)
Number of chronic illness, mean (SD)		1.6 (1.3)	1.24 (1.9)
**Health care used in last 3 months, n (%)**			
	General practitioner	811 (80.9)	537 (62.4)
	Gynecologist	677 (67.6)	504 (58.6)
	Traditional Chinese medicine	422 (42.1)	470 (54.7)
	Individual psychotherapy	566 (56.5)	109 (12.7)
	Family counselling	503 (50.2)	70 (8.1)
	Complementary and alternative therapy	657 (65.6)	585 (68)
	Sex therapy	377 (39.4)	36 (4.2)

^a^N/A: not applicable.

^b^Race data were not collected for the Chinese sample.

**Table 3:** Descriptive statistics.

Variables	United States			China			*t* test (*df*)^a^
	Value, mean (SD)	Participants, n	Value, mean (SD)		Participants, n		
Knowledge	14.58 (3.30)	1002	16.86 (2.49)		860	16.91 (1830)	
Perceived severity	14.84 (9.93)	1002	21.96 (12.38)		860	13.54 (1641)	
Perceived susceptibility	5.24 (1.69)	1002	4.56 (1.58)		860	–8.85 (1860)	
Perceived benefits	5.54 (1.41)	1002	5.74 (1.12)		860	3.44 (1849)	
Perceived COVID-19–related barriers	5.01 (1.97)	1002	4.15 (1.94)		860	–9.47 (1827)	
Perceived non–COVID-19–related barriers	4.80 (1.72)	997	2.94 (1.14)		860	–27.86 (1743)	
Self-efficacy	5.45 (1.40)	905	5.91 (1.09)		851	7.57 (1695)	
Motivation	5.58 (1.43)	919	6.03 (1.10)		835	7.45 (1710)	

^a^All *t* statistics were statistically significant (*P*<.001).

**Table 4:** Regression paths of the structural equations model with the United States and China data.

Regression paths	United States (n=1002)		China (n=860)	
	Unstandardized estimate (SE)	*P* value	Unstandardized estimate (SE)	*P* value
Knowledge → perceived severity	–0.69 (0.09)	<.001	–0.50 (0.17)	.003
Knowledge → perceived susceptibility	–0.09 (0.02)	<.001	0.01 (0.02)	.83
Knowledge → perceived benefits	–0.05 (0.01)	<.001	0.00 (0.02)	.86
Knowledge → perceived COVID-19–related barriers	–0.17 (0.02)	<.001	0.12 (0.02)	<.001
Knowledge → perceived non–COVID-19–related barriers	–0.17 (0.02)	<.001	0.02 (0.01)	.09
Knowledge → self-efficacy	–0.05 (0.01)	<.001	–0.02 (0.02)	.13
Knowledge → motivation	0.00 (0.01)	>.99	0.00 (0.01)	.93
Perceived severity → motivation	–0.01 (0.01)	.38	0.01 (0.00)	.03
Perceived susceptibility → motivation	0.03 (0.07)	.65	–0.03 (0.04)	.40
Perceived benefits → motivation	0.41 (0.06)	<.001	0.13 (0.03)	<.001
Perceived COVID-related barriers → motivation	0.08 (0.04)	.04	0.07 (0.02)	<.001
Perceived non-COVID-related barriers → motivation	0.02 (0.06)	.75	–0.35 (0.05)	<.001
Self-efficacy → motivation	0.40 (0.04)	<.001	0.43 (0.03)	<.001

## Discussion

### Principal Findings

Midlife women in the United States and China revealed rather different patterns of factors associated with menopausal help-seeking. For the US participants, greater knowledge conferred less perceived severity (indicated by bother about symptoms) but a lower self-efficacy to seek help. Furthermore, knowledge was related to lower perceived susceptibility, perceived benefits, and perceived barriers. In turn, motivation was only related to perceiving more benefits, COVID-19–related barriers, and self-efficacy, as was also the case among Chinese participants. For Chinese participants, higher knowledge was related to lower perceived severity and more concerns over contracting COVID-19 from attending clinics. In turn, greater perceived severity, perceiving more benefits, self-efficacy, and concerns for COVID-19, as well as lower non–COVID-19–related barriers, were related to greater motivation to seek help. Paradoxically for this sample, knowledge appeared to suppress the motivation to seek help by reducing perceived benefits, COVID-19–related barriers, and self-efficacy.

We put forward the following reasons that may explain why knowledge did not impose a direct positive effect on help-seeking motivation. First, a good proportion of menopausal women tend to rely on self-initiated lifestyle modifications rather than formal care for their menopausal symptoms [10,31]; thus, higher knowledge may have helped women tackle some menopausal symptoms on their own and effectively reduce help-seeking motivation. Second, Pimenta et al [47] found greater perceived control was related to fewer symptoms. Likewise, a systematic review by Ayers et al [48] revealed more reported symptoms in women with more negative attitudes toward menopause. If knowledge confers perceived control, it may reduce the experience of distress associated with menopause (which is supported by the negative relationships between knowledge and perceived severity in both of our samples), and eventually lead to lower help-seeking motivation. Third, greater menopausal knowledge may dilute menopause as something that should be tolerated, thereby reducing one’s propensity to seek professional help. In a qualitative study by Steffan [9], working midlife women in Britain exhibited strong self-reliance and personal responsibility either through putting up with or handling their menopausal symptoms and were silently “wanting yet not accepting help.” Knowledge may not only serve to normalize help-seeking behavior but may also dilute the intensity of the menopause experience. However, whether neoliberal individualization, or even trivialization, may have resulted in diminished service-seeking warrants more research into the lived experience of midlife women in different societies. In conclusion, whether knowledge imposes a direct effect on tackling the menopausal concerns or acts as an indirect effect via perceived control on palliating or diluting the distressing menopausal experience, which all in turn may reduce engaging in help-seeking, should be subject to further research.

Moreover, the role of knowledge on perceived barriers against help-seeking may be socioculturally dependent. In our Chinese sample, knowledge was related to perceiving more barriers against help-seeking, while the reverse was observed among the American sample. In a culture that emphasizes the importance of “face” and regards the discussion of the female body and sexuality a taboo [49], knowing more about menopause may mean learning more about the stigmatizing details of the health condition, which may in turn hinder help-seeking. Also, in China, since gynecological and menopausal well-being support as well as psychotherapies are not as common as in the United States, knowing more about menopause may be concomitant with large investments of time, money, and effort. Instead, Chinese medicine regards menopause as a manifestation of weakened “kidney” function that may be enhanced by herbal medicines and acupuncture [50], which serves as an explanatory model of a socially cohesive and culturally accepted mode of intervention. The barriers to seeking formal, “Westernized” or medical help could be amplified when self-care means are relatively accessible. Greater knowledge, however, may infer lower embarrassment and fewer financial, information, and time barriers in a society, like the United States, in which health systems have developed to embrace positive aging, femininity, and sexuality coupled with greater access to established menopausal care.

In terms of demographic factors, only being employed full-time and being married were associated with higher motivation to seek help in both samples. Menopausal state was also related to motivation to seek help, yet the directions of association were different in the 2 samples. While a full-time occupation and being married may confer financial and family support for help-seeking in both societies, other variables did not demonstrate cross-cultural consistency. An in-depth exploration of the demographic impetus of help-seeking is beyond the scope of this paper considering the limitations of our web-based survey panels. Social determinants of health, such as discriminatory and disabling beliefs about menopause, the accessibility of health care systems, the availability of treatments, social support, occupational characteristics, and general literacy, could be strongly related to the menopausal experience and may affect formal help-seeking [11]. Differences in cultural representations of the menopausal experience may also affect practitioners’ understanding of their patients’ distress and needs, especially with women from ethnic minorities [51]. Future studies may adopt a person-in-context approach to thoroughly explore microlevel, mesolevel, and macrolevel factors that influence menopausal help-seeking in midlife women.

### Limitations

In developing our survey, we needed to balance the need for a succinct measurement tool that engages the attention of our participants against the requirement for comprehensive coverage of the important constructs of the Health Belief Model. Therefore, most items were constructed specifically for this study. The widely used Champion’s Health Belief Model Scale has been adopted to examining the motivation for breast cancer self-examination, Papanicolaou tests, and other women’s cancer screening programs for women in menopause [45,52-54], but we found no adoption to the context of menopausal care to date. Also, as our survey relied on self-reported measures, the motivation to seek help can hardly be counter-checked with the actual help-seeking behavior.

Although the web-based survey panels, unlike face-to-face survey panels, have efficiently compressed the duration of data collection, which minimized the impacts of unexpected, time-sensitive factors (eg, sudden outbreaks), there are several limitations. First, we have no access to the number of potential participants our advertisements have reached, nor the exact randomization procedures of the panels with their eligible members. Thus, it is not possible to estimate the response rate. Also, although smart devices have become ubiquitous in the United States and China, web-based surveys have often resulted in the overrepresentation of individuals with higher socioeconomic status, those belonging to an ethnic majority, and those with a greater likelihood to have convenient and stable internet access. Our findings, however, could be particularly informative for telehealth development as we have engaged a group of women with good potential of early patronage [55]. Other factors, including distraction through concurrent apps on the smart device, self-selection bias, excessive nonnaivete, and social desirability bias, may have also affected the data quality of the web-based survey panels [56].

### Implications

Although our study was conducted during the height of the COVID-19 pandemic, our findings could be helpful for developing telehealth services targeting midlife women post the pandemic. Perceived COVID-19–related barriers were positively correlated with perceived severity in both of our samples. Higher levels of COVID-19–related concern may reflect either higher health consciousness [57,58] or an underlying health vulnerability that warrants an enhanced motivation to seek formal help. In this light, we call for expanding the use of telehealth to replace physical visits in menopausal care, especially for women who may be concerned about iatrogenic infection or during peaks of seasonal infectious diseases (eg, flu seasons). In fact, even prior to the COVID-19 pandemic, telehealth had been developed to reduce the direct and indirect costs of clinic attendance (eg, travel time and time off from work), as well as enhance privacy and autonomy by allowing consultation with practitioners at a location comfortable to the patient [59,60]. It is expected that these benefits will continue to prevail for a good proportion of patients even though some remote consultations may have reverted to face-to-face visits after the pandemic. Further research is needed to gather empirical evidence to indicate the proportion of remote consultations that have reverted to physical ones and which populations are likely to continue with remote consultations and telehealth services at-large post the pandemic.

Moreover, recent studies have reported that interventions including cognitive-behavioral–, behavioral-, or mindfulness-based therapies [61,62], walking programs supported by a virtual platform [63], and multidisciplinary clinics [64] may be useful for tackling menopausal symptoms as well as fostering the overall quality of life of women. As our study demonstrates, knowledge alone does not necessarily lead women to seek formal support. We suggest telehealth services focus on relieving psychosocial distress, connecting women to build a supportive and empathetic community, and enabling healthy lifestyles. AlSwayied et al [65] found early evidence that mobile-based physical activity programs are helpful for enhancing moderate to vigorous physical activities among midlife women. Another systematic review by Sediva et al [66] on 13 digital health interventions found that most interventions for midlife women targeted weight loss, lifestyles, and menopausal symptom management and primarily relied on the provision of instructions on healthy behaviors, fostering capability, and motivation. Although the heterogeneity of the studies and their outcomes forbids a conclusion about the general effectiveness of these digital health interventions, it is hoped that these contents may build confidence and familiarity in women with their health care providers, thus connecting women in need to more specialized support. Telehealth may be used to facilitate shared decision-making in the treatment of menopausal symptoms. The North American Menopause Society has developed MenoPro, an app that relies on a menopause decision-support algorithm, which can be accessed by both patients and health care providers to make decisions for pharmacologic or nonpharmacologic treatments [67]. The app may support a more transparent and personalized approach for risk stratification and shared decision-making that in turn enhances perceived benefits and self-efficacy of the support, which are 2 facilitators for help-seeking revealed in our findings.

A multistakeholder, participatory design study conducted by Backonja et al [21] found that women with menopause had distinctive concerns compared to their health care providers when it came to their desired Menotech functionality, with the former wanting Menotech to “understand, prevent, and positively reframe their menopause experience” and the latter placing greater emphasis on “tracking and patient-provider communication.” Future Menotech is expected to go beyond symptom tracking and assemble the collected data to predict the onset of bothersome experiences and prevent such experiences by offering personalized, quotidian suggestions, which, in turn, integrate and celebrate this life transition under the expanding occupational lifespan of midlife women. Our study points to the intricate cross-cultural differences in the role of knowledge between American and Chinese midlife women. In this light, we call for the development and testing of culturally targeted Menotech services that may enhance perceived benefits and self-efficacy or even capitalize on the health consciousness of midlife women to foster help-seeking. These culturally sensitive considerations may span from content (eg, inclusion of indigenous therapies, such as Chinese medicine and Qigong, among others) and service mode (eg, collaboration with established telehealth platforms of general practice vs a standalone system) to technologies (eg, apps or devices) and policies about data protection and privacy.

### Conclusion

This study is the first to compare the factors affecting help-seeking tendencies for menopausal care among midlife women in the United States and China. Our findings reveal disparate pathways of help-seeking. Higher self-efficacy, perceived COVID-19–related barriers, and perceived benefits, but not knowledge, were related to higher help-seeking motivation in both samples. In the Chinese sample, motivation to seek help was associated with higher perceived severity and lower perceived non–COVID-19–related barriers. In the US sample, knowledge had negative associations with all factors. Implications for post–COVID-19 telehealth services for menopausal support have been discussed.

## Abbreviations

CFI: comparative fit index
MTurk: Amazon Mechanical Turk
RMSEA: root-mean-square error of approximation
SEM: structural equations model
SRMR: standardized root-mean-square residual
TLI: Tucker-Lewis index
